# The effect of durability of knowledge transfer through reminiscence on the moral sensitivity of nurses

**DOI:** 10.1186/s12912-022-01060-4

**Published:** 2022-11-09

**Authors:** Farideh Bahrieni, Parviz Azodi, Abdollah Hajivandi, Zahra seddighi, Faezeh Jahanpour

**Affiliations:** 1 Nursing and Midwifery faculty, Bushehr University of Medical Sciences, Bushehr, Iran; 2 Paramedical Faculty, Bushehr University of Medical Sciences, Bushehr, Iran; 3 Health Faculty, Bushehr University of Medical Sciences, Bushehr, Iran; 4 Persian Gulf martyrs hospital, Bushehr University of Medical Sciences, Bushehr, Iran

**Keywords:** Professional ethics, Nurses, Moral sensitivity, Reminiscence

## Abstract

**Background:**

Nursing is a moral attempt and endeavor. Moral sensitivity training is one of the most important ways to strengthen nursing moral decision making. Thus, the purpose of this study was to determine the effect of durability of knowledge transfer through reminiscence on nurses’ moral sensitivity.

**Methods:**

The present study is a controlled randomized clinical trial conducted in pre- and post- interventional forms. The research population consisted of all nurses working at Bushehr Persian Gulf martyrs training hospital in 2017, 68 nurses participated as samples in the study selected by available sampling method and then randomly assigned to two groups of intervention and control groups. In this study, Morphological Sensitivity Questionnaire of Lautzen was used. Validity and reliability of the questionnaire have been confirmed. The pre-test was obtained from the control and intervention group. An 8-hour reminiscence training session was held for the intervention group. In order to study, the durability of training, posttest was obtained from both groups. Data were analyzed by SPSS software version 19 and using t-test, Chi-square, paired t-test.

**Results:**

The mean moral sensitivity score in nurses before intervention in both groups was not statistically different (P = 0.42). The comparison of the mean scores of nurses’ moral sensitivity after intervention in the control and intervention groups, which in fact, indicates the durability of education, showed a significant difference. (P < 0.001).

**Conclusion:**

The results of this study showed that the transfer of knowledge and education through reminiscence has a lasting effect on enhancing the moral sensitivity of nurses. Therefore, its inclusion in nursing educational programs as a training strategy can be considered as a step toward facilitating the training of nurses’ moral sensitivity.

**Trial registration:**

This study is registered by Iranian Registry of Clinical Trials with decree code: IRCT2016021612830N18 on May 17, 2017. This study adheres to the CONSORT 2010 statement guidelines.

## Background

Nursing is a moral endeavor, and nurses are the largest provider of health care services and have a significant impact on the quality of health care. They face many challenges and problems due to their professional nature [1]. One of the issues that plays a fundamental role in moral decision-making is moral sensitivity [2] In other words, moral sensitivity is a prerequisite for moral behavior and judgment [3].

Despite the emphasis on the need to pay attention to ethics in all phenomena, what is being seen is the skepticism about the professional competence of nurses for decision-making, which has been criticized many times through the health system and outside it [4]. The moderate level of moral sensitivity in nursing students has been reported by Yeom et al. (2016)[5]. In Iran, studies have also been conducted to assess the moral sensitivity of nurses and reported different results. Some researches stated that nurses were weak in using moral principles in their decisions [6]. Some other studies have shown the moral sensitivity of nurses in the treatment and care of patients at the moderate level [7].

Public concerns about moral health issues are increasing and this make it an undeniable necessity to address the moral category. Failure to deal with moral problems leads leaving nursing job by 20% of nurses or they want to change their place of work [8], moral controversies are the source of psychological stress and burnout of nurses [9]. On the other hand, the development of moral sensitivity leads the nurse to take steps to do the patient, and has a better management in the clinical environment, it also makes good use of moral codes and reduces stress [10, 11].

Given the importance of the subject and the results of previous studies, it seems that the adoption of measures to assign a special place to ethics education is essential in nursing education programs [12], because 67% of nurses considered moral education as inadequate in official education [13] and introduced the lack of ethics education as an obstacle to the development of morality in nursing and moral sensitivity [14].

Although recent attention has been paid to nursing ethics education, however, there is still not much information about the teaching of ethics, methods and training patterns that enhance the competence of nurses [15]. Researchers believe that traditional methods of moral education, which rely on principles, rules, theories and moral codes do not necessarily prepare nurses for moral decision-making in the clinic and the debate continues to select the best method for teaching nursing ethics[16].

Effective, deep and efficient learning, maintaining and increasing motivation and above all information durability in the minds of students have always been a problem for educators, and in this regard; one of the best solutions to solve this problem is the use of educational methods that create more durability of information in the minds of students [17].

One of the training methods is the expression of experiences and memories. The memories of individuals reflect the activities and realities and the level of social interaction of individuals and the use of personal memories of individuals is a way of learning from past experiences and subjective knowledge of individuals. Learning through reminiscence and expressing experiences is a kind of interactional learning. The purpose of indecision is thinking on an experience purposefully and returning to thoughts and reminding thoughts and memories of an issue, thinking consciously, and taking into account all the aspects and backgrounds of that incident, and ultimately trying to find a new solution of changing it that position, if required. Individual stories can express the challenges and conflicts in moral issues in a human form, using the power of expressing strong emotions and interpersonal connections in their nature[18].

Recounting experiences by being in a group provides a situation for learners to rethink and reflect. Interaction and communication in the group is a factor affecting creativity and learning. In addition, with the help of this method, the experiences of learners are shared with each other, and with collective rethinking, the possibility of obtaining new solutions and perspectives will increase. Recounting experiences by being in a group provides a situation for learners to rethink and reflect. Interaction and communication in the group is a factor affecting creativity and learning[19].

Studies have been done on the impact of education on moral sensitivity. In the study of Imanifar et al. in 2015 in Birjand, it was found that, two training methods for narration and lectures promote the moral sensitivity of nurses [12].

Also in a semi-experimental study conducted by Borhani et al. in 2012 in Kerman, it was found that all dimensions of moral sensitivity increased after the training by the workshop and follow up [20]. The results of Kim’s experimental study in 2014 indicated that, a hybrid learning program that included case-based learning with problem-based learning had a positive impact on the development of moral values ​​in undergraduate nursing students [21]. Also, the results of another experimental study conducted by Baykara in Turkey in 2015, suggests that ethics training and counseling services increase moral sensitivity [22]. Experimental study by Yeom et al. in 2016 in Korea was conducted on 70 undergraduate nursing students from Seoul University. The results showed that after training, the level of patient care, the range of moral sensitivity, awareness and critical thinking significantly improved [5]. The quasi-experimental study of Choe et al. in 2014 was conducted on 93 nursing students at a nursing college in South Korea. Two teaching strategies, active learning and discussion were used for teaching ethics. After ethics training, students’ understanding of moral issues was improved in both classes and active learning and discussion training methodology were identified as two effective methods for recognizing nursing students of moral issues [23].

In all of the above studies, training methods have been described as effective ways of learning the professional ethics of students and nurses. In the review of the literature, the study of professional ethics in the form of reminiscence has not been found. Therefore, considering the importance of the issue of ethics in nursing, the role of indirect education in behavioral sciences and the need for research based on new educational methods and the assumption that the use of the method of expressing memories in the teaching of moral concepts and principles in the field of medical science can lead to valuable results. And the lack of empirical studies that documented the effect of this method, and inadequate teaching of moral issues to nurses and the lack of satisfaction of people with respect to moral principles by nurses, we decided to conduct a study to determine the effect of durability of knowledge transfer through reminiscence on nurses’ moral sensitivity.

## Implementation method

### The aim and design of the study

The study is a randomized controlled trial (RCT) designed to determine the effect of durability of knowledge transfer through reminiscence on the moral sensitivity of nurses working in Persian Gulf Martyrs Teaching Hospital in Bushehr. This study was conducted pre, post and one month post the intervention. This report was written according to the CONSORT guideline. The study adheres to the Consolidated Standards of Reporting Trials [CONSORT] [24]. Data were collected between August 2017 and January 2018.

### Study population and sample

The study population consisted of all nurses working in the Bushehr Persian Gulf martyrs training hospital (295 person). Since the main goal is to compare the mean scores of moral sensitivity in both the control and intervention groups, based on the formula *N* = [(4σ^2^) (*Z*_(1−(α/2))_ + *Z*_(1−ß)_)^2^] ÷ *E*^2^ (1-β = 0.8 power of test, α = 0.05 error of test, Z_1−β_=0.84, Z_1− **α/2**_ = 1.96) with a standard deviation of 12, and a difference diagnosis of 8 scores between the two groups (according to previous similar studies)[25], the test power was 80% and the test type error was equal to 5% of the 68 samples. Therefore, 68 nurses participated in the study, who were selected by available sampling method. Then 34 samples were randomly assigned to each intervention and control group.

**The inclusion and exclusion criteria**: inclusion criteria of the study include: 1- Having a minimum undergraduate degree in nursing 2- Having at least one year of clinical work experience. Exclusion criteria include: The history of participation in a workshop or similar training courses.

### Data collection tool

A questionnaire was used in this study, which included two parts. The first part of the questionnaire includes demographic information (age, gender, marital status, work record, degree of education). In the second part, Moral Sensitivity Questionnaire (MSQ) in decision making was used to assess the moral sensitivity of the nurses, this questionnaire was developed by Lützén et al. in Sweden [26]. Then it was used in different countries including Iran. This questionnaire consists of 25 questions and measures the status of nurses’ moral sensitivity in clinical presentation and the score for each question is obtained in the Likert method as completely agree (4), fairly agree (3), fairly disagree (2), completely disagree (1) and neutral (0). The highest score is 100 and the lowest is zero. Accordingly, if the total score of each sample is between (0–50), it has low moral sensitivity, (50–75) with a moderate moral sensitivity and (75–100) with high sensitivity [27].

### Validity and reliability of the Tool

The reliability of this questionnaire has been studied in several stages according to Hassanpour et al. (2011) so that for the validity and reliability of the tool, the questionnaire was first translated from English to Persian according to the standards of the World Health Organization (translation of questionnaires) and according to the cultural conditions of Iran. Then the translation was returned to the original language and matched with the original text. For formal and content validity as well as to ensure the correct translation, the questionnaire containing the translated text along with its English text was given to 10 expert professors. In addition, the appropriateness of each of the questions with the objectives of the research was determined, and after collecting the data, the validity of each question was obtained, and after the suggested changes and corrections, the validity of the questionnaire was confirmed. The reliability of the questionnaire was calculated by him in a pilot study on 20 nurses, after collecting and checking the internal consistency of the questionnaire, its analysis was calculated with Cronbach’s alpha coefficient, which was 0.81 and confirmed [27]. Also, the reliability of the questionnaire was calculated to be 0.80 in Izadi et al. research (2013) after the collection of data of 20 nurses, internal consistency with Cronbach’s alpha coefficient [7]. The reliability of the questionnaire in the United States was 0.76 [28] and in Korea it was 0.78 [29].

### Randomization and blinding

All participants were randomly divided (ratio 1:1) into the intervention or control group. Randomization was done with computer random numbers. From the research team, Z.S enrolled participants, and F.B assigned participants to intervention and control group. A.H performed the statistical analysis.

It is not possible to mask the intervention in this type of trials. To avoid any bias, data entry and analysis will be performed by neutral researchers who are blinded to group allocation.

### Method of implementation

The research environment in this study was Persian Gulf Martyrs Teaching Hospital in Bushehr city. After approval of the research project at the research deputy of the Bushehr University of Medical Sciences and obtaining a legal and moral license, and presenting the Ethics Committee’s Letter to the Bushehr Persian Gulf martyrs hospital, an intervention group was invited to a session and the study objectives were explained to them. At the end of the meeting, the informed consent form and the pre-test were obtained. At another session, the control group was invited and the study objectives were also explained to them and the informed consent form and pre-test form were also obtained from the control group. The intervention group was divided into 4 subgroups and an 8-hour reminiscence session (in one day, morning and evening) was held for each subgroup, each intervention group received 8 h of training. During the meetings, the nurses volunteered to tell the memories of the events which has been about professional ethics issues and they themselves participated actively or witnessed that event. At each meeting, there were two experienced experts who analyzed the memories and, if necessary, provide the right solution. For the control group, the reminiscence session was not formed. After the end of the intervention, the post-test was taken from both groups. To evaluate the durability of the training, a post-test was taken one month later from the intervention and control groups.

### Data analysis method

Data collected by questionnaire were coded and analyzed by SPSS software version 19, after entering the computer. Data were analyzed using descriptive statistics and t-test for comparing two groups in terms of quantitative demographic variables such as age and work experience. Chi-square test was used to compare the two groups in terms of qualitative demographic variables such as gender. T-test was used to compare the mean scores between two groups and paired t-test to compare the pre and post-intervention mean score in each group. To compare the mean scores of moral sensitivity during the study period in the intervention and control groups, which in fact determines the durability of training, repeated measures analysis of variance test was used. P < 0.05 was considered statistically significant.

## Results

Comparison of demographic information in two groups showed that the two groups were similar and there is no significant difference (Table 1).

**Table 1:** Determination and comparison of demographic information in two groups of test and control

Demographic factors		Intervention Group	Control Group	P value*
Age	M ± SD	33.08 ± 5.41	32.85 ± 5.05	P = 0.85 t = 0.29
Sex	Man	20.6% (N = 7)	20.6% (N = 7)	P = 1 x ^2^ = 0
	Female	79.4% (N = 27)	79.4% (N = 27)	
marital status	Single	38.2% (N = 13)	26.5% (N = 9)	P = 0.43 x ^2^ = 0.57
	Married	61.8% (N = 21)	73.5% (N = 25)	
Education	Bachelor Degree	100% (N = 34)	94.1% (N = 32)	P = 0.49 x ^2^ = 0.47
	Masters	0% (N = 0)	5.9% (N = 2)	
work experience	M ± SD	9.6 ± 4.68	9.16 ± 5.25	P = 0.71 t = 0.37

M Mean, SD Standard Deviation.* p < 0.05

Chi-square test was used for variables of gender, marital status and education.

T test was used for variables of age and work experience.

The mean score of moral sensitivity of nurses before intervention in the two groups was not statistically significant and both groups were identical. Comparison of mean scores of moral sensitivity after intervention in the control and intervention group was obtained by independent t-test and there was a significant difference between the two groups. Also, comparison of mean scores of moral sensitivity before and after the intervention in the control and intervention group was obtained by using paired t-test. Comparison of mean scores of moral sensitivity before and after the intervention was significant in the intervention group and was not significant in the control group (Table 2).

**Table 2:** Comparing the mean scores of moral sensitivity between the two groups and in each group

group	Intervention (M ± SD)	Control (M ± SD)	P value*
Before intervention	71.0 ± 9.92	69.44 ± 5.32	P = 0.42 t = 0.81
After intervention	84.29 ± 8.63	70.02 ± 5.23	P < 0.001 t = 3.32
P-value*	P < 0.001 t = 3.32	P = 0.07 t = 1.82	

M Mean, SD Standard Deviation.* p < 0.05

T-test was used to compare the mean scores between two groups and paired t-test to compare the pre and post-intervention mean score in each group.

To compare the mean scores of moral sensitivity during the study period in the intervention and control groups, which in fact determines the durability of training, repeated measures analysis of variance test was used, and the results showed that the mean changes in moral sensitivity scores during the study group in the experimental group Comparison with the control group had a significant difference (Table 3).

**Table 3:** Comparison of the mean scores of moral sensitivity during the study period in the test and control groups

group	Intervention (M ± SD)	Control (M ± SD)	
Before intervention	71.0 ± 9.92	69.44 ± 5.32	
After intervention One month after intervention	84.29 ± 8.63 84.91 ± 7.87	70.02 ± 5.23 69.85 ± 5.59	
P-value*	P < 0.001 F = 5.53	P = 0.135 F = 2.31	

M Mean, SD Standard Deviation.* p < 0.05

ANOVA test was used for compare the mean scores during the study period in the intervention and control groups.

## Discussion

The present study was conducted with the general purpose of “determining the effect of durability of knowledge transfer through reminiscence on the moral sensitivity of nurses”. Findings from comparison of demographic factors between intervention and control groups showed that, the two groups did not have significant difference in terms of age, gender, marital status, education, and work experience, and were homogeneous.

Based on the obtained results, the average moral sensitivity score between the two intervention and control groups at the beginning of the study was at an average level. Farasatkish et al. study (2014) showed that the average moral sensitivity score of nurses was in the medium range [30]. In South Korea, psychiatric nurses had moderate moral sensitivity [31], which is consistent with the results of this study.

Karimi et al.(2016) have stated that the level of moral sensitivity in nursing students and nurses has reached an optimal level [32] also, Mousavi et al. have reported that the mean score of moral sensitivity of nursing students and nurses is higher than average [33]. However, in the study of Imani et al. (2017), the mean score of moral sensitivity among nurses was reported to be low [34], which is not consistent with the results of the present study. The difference in the results can be caused by the level of culture and the study environment and the atmosphere of the work environment.

Comparison of mean scores of nurses’ moral sensitivity before intervention in the intervention and control groups showed that, the mean score of nurses’ moral sensitivity before intervention in the two groups was not statistically significant. The mean score of nurses’ emotional sensitivity after intervention was compared in the experimental and control groups. There was a significant increase in the mean score of moral sensitivity after intervention in the intervention group compared to the control group.

In the review of the literature, no study on the teaching of professional ethics by reminiscence has been found. But these findings are consistent with findings provided by some researchers. For example, in a study by Choe et al. (2014) entitled “The Effect of Structural Teaching Methods on Ethics Education for Nursing Students: A Semi-Experimental Study”, it was found that, knowledge, skills and moral competence of students after ethics education have been improved through active education and group discussion [23].

This finding is also consistent with the results obtained by Baykara’s (2015), which showed that ethics education increases student awareness of moral violations in hospitals and it helps them to have better interventions and care in this field. In this study, the researcher believes that moral education through discussion can have a positive impact on their emotional and cognitive outlook, given the cases experienced by the students in the hospital [22]. Imanifar et al. (2015) have shown that the two methods of narrative ethics and lecture have significantly improved the moral sensitivity of nurses in each group compared to before the intervention, which is consistent with the results of the present study[12].

But this finding is in contradiction with the study of Hough (2008). He considers the small effect of experience and education on sensitivity in moral decision-making in his study and states its reason as, some moral problems make the nurse confused and prevent the nurse to make good decisions [35]. This contradiction appears to be due to the following reasons. The first is the type of study, because the research has been qualitative in the above study, while this study was conducted as a controlled clinical trial. In the Hough’s study, the nurses working in the Intensive Care Unit were interviewed and the interview method has been a rethinking of past moral decisions, while in this study nurses from all wards were participated in the study and randomly divided into two groups of test and control. Also, inconsistent with the results of the previous research, Yeum et al. (2017) reported that there was no change in the overall scores for moral sensitivity and critical thinking [5]. Different results it can be caused by a different educational method.

The mean of moral sensitivity scores before and after intervention in the test and control group was calculated and compared and, it was found that the mean scores of the moral sensitivity before and after the intervention in the test group was significant and in the control group it was not significant which shows the positive effect of training moral sensitivity with the reminiscence method in the intervention group. This finding is consistent with the study of Tamimi et al. (2013) showing that, the transfer of tacit knowledge through the narrative expression of individual experiences improves the clinical competence of nurses in the Intensive Care Unit of the Shahid Rajaee Cardiology and Research Center for Research and Therapy in order to communicate effectively with the patient and the treatment team [36].

Kim’s experimental study (2014) was conducted on 71 undergraduate nursing students and it was found that, the score of moral values in​​ students after combination learning including case-based learning with problem-based learning, has significantly increased that, the result of this study is consistent with this result [21].

Also, the results of Ahns study (2014) in Korea showed that training ethics principles significantly improved the level of patient care, the range of moral sensitivity, the level of students’ knowledge and critical thinking. The question and answer about moral issues and expressing different perspectives and discussing these cases, actively promotes critical thinking, which is also consistent with the present study [37].

To compare the mean scores of moral sensitivity during the study period in the intervention and control groups, which in fact determines the durability of training, repeated measures analysis of variance test was used, and the results showed that the mean changes in moral sensitivity scores during the study group in the experimental group Comparison with the control group had a significant difference. The results of the present study show the effect of the intervention designed based on reminiscence; therefore, in addition to being effective, the educational method has also had a lasting effect. This finding was consistent with the results of Ghasemi (2012) and Tamimi (2013). In these two studies, the effect of the storytelling expression of clinical experiences in the course of workshops on the competence of nurses was assessed. Findings from post-test one month after intervention showed that, the educational method of expressing experiences through storytelling leads to the improvement of nurses’ clinical competency skills [36, 38]. Also, in Jamshidian et al. study (2017), the average score of moral sensitivity increased significantly immediately and two months after the intervention [39]. The results of these studies are in line with the recent study.

However, in the study of Imanifar et al. (2014), the average score of moral sensitivity three months after the intervention showed a significant decrease compared to immediately after, in two groups [12], which is not consistent with the results of the present study. The decrease in the average score of moral sensitivity in Imanifar et al.‘s study may be due to a different teaching method that has less durability. Also, in the current study, the durability of education was measured one month after the intervention, while in Imanifar’s study, the duration was longer and it was measured three months after the intervention.

Also, this finding of the present study is different and inconsistent with Mayhew’s results (2009). The results of the aforementioned study have shown that, with the passage of time and through training courses, in some cases, no significant changes in moral sensitivity and subsequent development of moral behavior have been observed [40]. This contradiction seems to be due to the following reasons. First, the study samples are in the above study include accounting students, but in the present study, the samples included nurses working in the hospital environment, the educational method in the study was not an active participation of students and samples have received the educational program, while in the present study, the samples have actively expressed and shared the memories of their work experience which can have a positive impact on the learning and promotion of their moral sensitivity. Also the area of ​​moral sensitivity in the Mayhew’s study has been financial reporting and financial issues but in the present study, the area of moral sensitivity of the patient care and communication has been evaluated.

## Limitations

The limitation of this study is that both the intervention and control groups worked in a hospital and there was the possibility of information exchange between the two groups, therefore, in order to reduce this limitation, the researcher asked the intervention nurses not to share the memories expressed in the training class with their other colleagues. Also, informed consent and pretest in separate sessions were obtained from both groups.

## Conclusion

The results of this study showed that the transfer of knowledge and education through reminiscence has a lasting effect on enhancing the moral sensitivity of nurses. Therefore, its inclusion in training programs for nurses as an educational strategy can be a step toward to facilitate moral training for nurses.

## Data Availability

The datasets used and analyzed during the current study are available from the corresponding author on reasonable request.

## List of abbreviations

**MSQ**: Moral Sensitivity Questionnaire
**RCT**: Randomized Controlled Trial
